# Tumour measurements on imaging for clinical trial: A national picture of service provision

**DOI:** 10.1038/s44276-025-00131-8

**Published:** 2025-03-27

**Authors:** Georgina Hopkinson, Jonathan Taylor, Jonathan Wadsley, Angela Darekar, Christina Messiou, Dow-Mu Koh

**Affiliations:** 1 Department of Radiology, The Royal Marsden NHS Foundation Trust, London, UK; 2 Sheffield Teaching Hospitals NHS Foundation Trust, Sheffield, UK; 3 Weston Park Cancer Centre, Sheffield, UK; 4 University Hospital Southampton NHS Foundation Trust, Southampton, UK; 5 Division of Radiotherapy and Imaging, The Institute of Cancer Research, London, UK

## Abstract

**Background:**

Radiological response evaluation metrics such as RECIST 1.1 inform critical endpoints in oncology trials. The UK was the 6th highest recruiter into oncology trials worldwide between 1999 and 2022, with almost 9000 oncology trials registered during the same period. However, the provision of tumour measurements for oncology trials is often ad hoc and patchy across the NHS. The aim of this work was to understand the barriers to providing an effective imaging tumour measurement service, gain insight into service delivery models and consider the successes and challenges from the perspective of both service providers and end users.

**Methods:**

An electronic survey was distributed to those who provide tumour measurement response review for clinical trials (service providers) and those that request and use such measurements in trial activities (service users).

**Results:**

Responses from 35 sites demonstrated substantial variation in service provision across the UK. Despite workforce pressures, service is largely delivered through radiologists with a minority utilising radiographer role extension. Only 20% of the service providers had dedicated training and 29% received robust financial reimbursement.

**Discussion:**

Service variation is likely a consequence of limited training, education and infrastructure to support robust service, compounded by increasing radiology workload and workforce pressures.

## Background and local perspective

Integral to the evaluation of the efficacy of novel cancer therapeutics is the assessment of disease burden on radiological imaging, where changes in the number and size of tumours defined by established criteria, such as the Response Evaluation Criteria in Solid Tumours (RECIST 1.1) [1], are used to determine tumour response to treatment. RECIST is used from exploratory to phase III commercial drug trials to determine best response and the time to events such as progression free survival/overall survival [2]. Other response criteria, such as iRECIST to assess immunotherapy agents, mRECIST for mesothelioma/ hepatocellular carcinoma and Choi criteria for gastrointestinal stromal tumours also have specific clinical applications [3–5].

The use of RECIST 1.1 and other associated criteria provide objective endpoints and standardised outputs for clinical trials. However, inter-observer variability in applying these criteria is dependent on reader experience for image interpretation and analysis [6, 7]. Hence, imaging tumour measurements for clinical trials are traditionally undertaken by radiologists with relevant understanding and experience. However, the radiology workforce is in crisis and the UK now has a 29% shortfall of clinical radiologists which is projected to rise to 40% in 5 years [8]. At present, only 24% of radiology department clinical directors believe they have sufficient numbers of consultant radiologists to deliver safe and effective routine clinical care [8]. Providing patients access to clinical trials, including the necessary tumour measurement services to facilitate this, is a core aspect of high-quality cancer care. When departments are under such extreme pressure, this work can be perceived to be of lower priority and not considered to be part of the core clinical service. This can result in sites not recruiting to oncology studies, denying patients treatment options.

In this paper, we aim to establish a better understanding of the provision and utility of tumour measurements for clinical trials nationally by means of an online survey directed to participants of previous RECIST courses organised by the co-authors. The feedback at these courses identified the need for further multidisciplinary training and development, but also revealed geographic variations in the delivery the imaging assessments.

## Method

An electronic survey tool was designed by a multi-disciplinary team, including radiologists, a radiographer and a clinical scientist, all with extensive experience of providing tumour measurements for clinical trials as well as an oncologist with substantial experience requesting and interpreting tumour measurements for clinical trials as a primary investigator in oncology research. The survey was reviewed locally for readability and ease of completion prior to distribution.

The sample population criteria required participants to work for an institution that undertakes oncology clinical trials that require imaging assessment using RECIST criteria or similar (including but not limited to iRECIST, mRECIST, RANO, Lugano, Cheson). The survey required the participant to choose their role, as either a ‘RECIST service user’ or ‘RECIST service provider’ to allow the completion of the appropriate set of questions. Multiple responses were permitted to some questions where appropriate.

The survey was open for 3 months from January to March 2024 and was distributed to both those who provide tumour measurement response review for clinical trials (service providers) and those that request and use such measurements in trial activities (service users). Distribution was via established NIHR mailing lists, directly to the attendees of previous and planned educational meetings held for imaging tumour measurements in clinical trials and amplified through local networks and via social media.

The responses have been reviewed and analysed looking for trends and patterns using descriptive statistics (number of responses and percentages).

## Results

A total of 69 responses were received. Thirty-four responses from 21 sites were received from service providers and thirty-five responses from 23 sites were received from service users. Allowing for multiple responses from the same institution, total of 35 different Trusts/institutions were represented across both groups. Distribution of responses by role of responder is detailed in Table 1.

**Table 1:** Distribution of responses by role of responder.

Responses by role	Number of responses	Percentage
**Service providers: Total 34 responses from 21 sites**		
Radiologists	29	85%
Diagnostic Radiographers	3	9%
Clinical oncologists	1	3%
Unknown	1	3%
**Service users: Total 35 responses from 23 sites**		
Consultant Oncologists	6	17%
Principle Investigators	4	11%
Research nurses	11	31%
Other trial staff	12	34%
Research fellow	1	4%
Unknown	1	4%

**Fig. 1:**
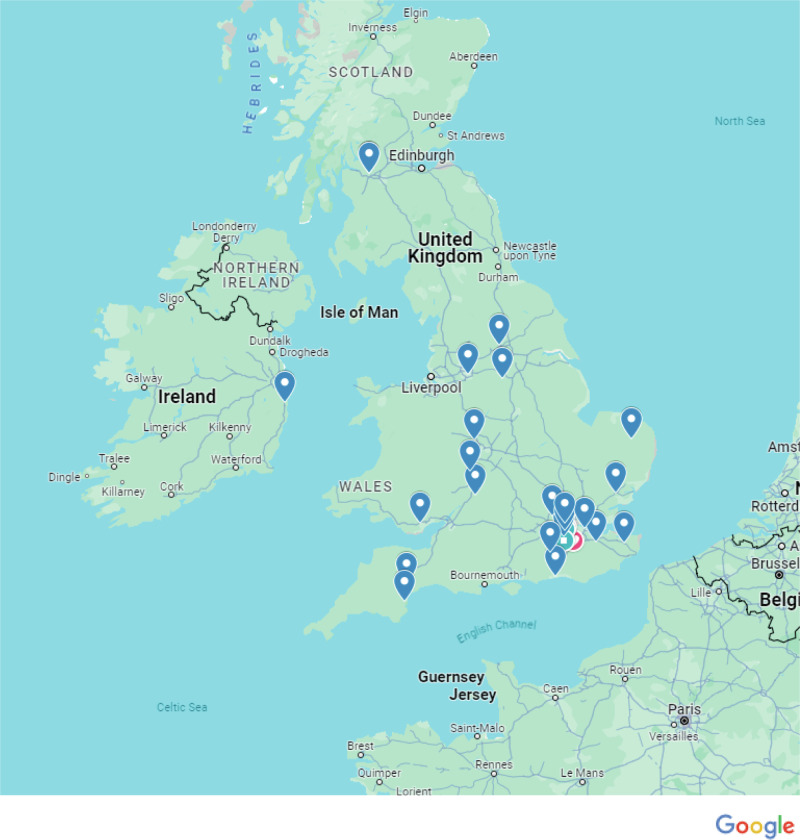
Distribution of responses from service users.

**Fig. 2:**
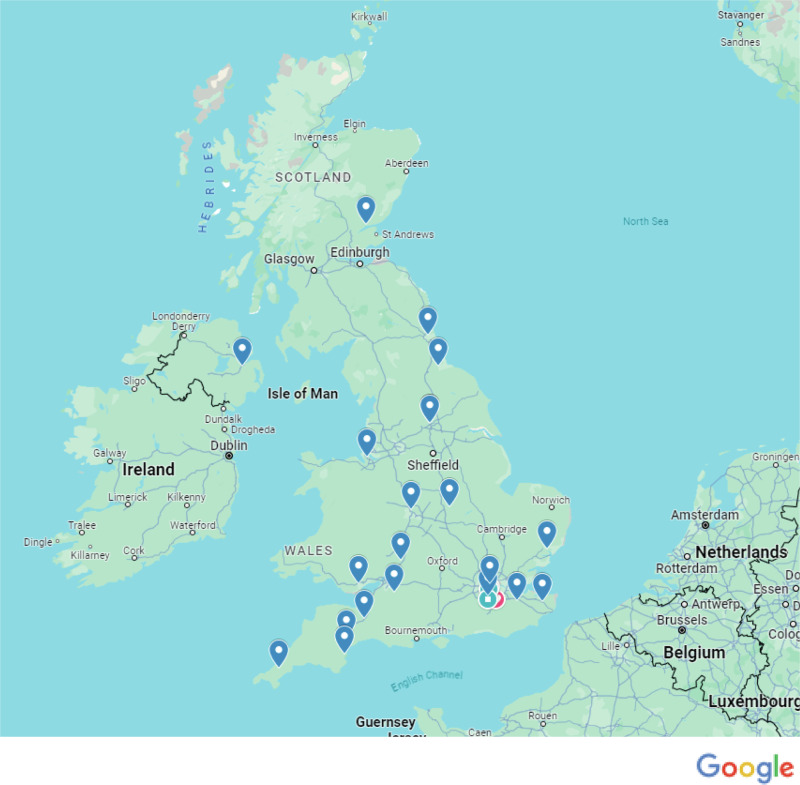
Distribution of responses from service providers.

The geographic distribution of responses from service users and providers are demonstrated in Figs. 1 and 2 respectively. Whilst there was good geographical distribution of responses achieved, with responses from sites across the country and indeed the UK, no pattern was recognised between the quality of the tumour measurement service and geographical location.

Questions and findings that were recorded from both service providers and service users are as follows:

### Q: Who currently performs the ‘RECIST’ assessments at your institution? (tick all that apply)

26 institutions out of 35 (74.2%) answered radiologists exclusively (consultant or trainee). 5 sites out of 35 (14.2%) also use diagnostic radiographers and 3 sites out of 35 (8.5%) reported the work being undertaken by non-radiology clinicians and other research staff in addition to radiologists, including one site that outsources the work to a private company. One site is not able to offer a ‘RECIST’ service due to lack of capacity.

### Q: Is there any infrastructure for the delivery of a service to provide tumour measurements for clinical trial at your institution? (Free text response)

Seven out of 35 sites (21%) reported no established infrastructure, whilst 6 sites (17%) had dedicated teams. One site reported ‘trial unit funded sessions for consultant radiologists’ and another unit ‘funded programmed activity (PA) time in radiology from commercial trial income’, one site ‘paid radiologist bank hours’, one had it included into their job plan. One site outsourced to an ‘external reporting agency’ for commercial trial measurements. The remaining sites that responded relied on either an individual or a small pool of interested radiologists who could be approached on an ad-hoc basis to undertake the work. There were reported benefits from a *‘co-ordinator who tracks trial set-up, patient identification, scanning workload and funding allocation’*.

In the following sections, we report on the feedback from service providers and service users respectively.

## Service providers’ perspective

### Q: Does the individual undertaking the ‘RECIST’ assessment have specific training for this role? (tick all that apply)

A: Forty responses were received from 34 respondents. Eight (20%) received either accredited or in-house training. 13 (32.5%) respondents reported completing a period of shadowing/supervision. Four (10%) respondents confirmed that they had received no specific training. 8 (20%) respondents were unsure of the training undertaken, whilst 7 (17.5%) selected ‘other’ with free text including ‘*attendance at scientific meetings’* and *‘variable dependant on the radiologist’*.

### Q: Is the service and/or measurements subject to audit?

A: Thirty-four responses were received from 34 respondents. 4 (11.7%) reported a regular audit programme, 1 (3%) reported ad hoc audit, 3 (8.8%) reported no current audit but plans to in the future, 16 (47%) reported no current audit in place and no plan to implement one. 10 (29.4%) selected ‘other’, enabling a free text response in which two respondents highlighted that measurements for clinical trial are *‘often reviewed by external trial monitors or sponsors’* and felt any additional audit was not required.

### Q: If the ‘RECIST’ service is performed by radiology, is there a method for financial re-imbursement to radiology?

A: Thirty-four responses were received from 34 respondents. Ten (29%) confirmed there was financial re-imbursement, 4 (12%) no re-imbursement, 13 (38%) were unsure. One site reported being unsure if any payment was received, whilst another reported it being *‘reflected in reporting allocations”*. Two responders reported this being variable between trials. Three sites reported using trial income to fund radiologist sessions including additional hours via the hospital bank.

### Q: Are the results generated using dedicated software?

A: Six (18%) responders did not answer, 25 (73%) did not use dedicated software and 3 (9%) respondents indicated use of a PACs-based or locally developed solution.

### Q: Are you happy with the level of service you are currently providing?

A: Seventeen of 34 (50%) respondents answered ‘we could do better’, 13 (38%) were happy, 1 (3%) responded that their service needed a lot of work and 3 (9%) selected ‘other’, enabling a free text response including *‘excellent support from radiographer and admin staff’*.

### Q: Is there anything you would like to see developed or improved in the provision of this service at your institution in the future? (free text response)

A: Of the 34 responders, 5(14.7%) did not answer, 4 (11.7%) felt that there was nothing they would like to see developed, 8 (23.5%) mentioned a need for additional training, 10 (29.4%) described an ongoing need for investment in capacity, and sustainability of service. A request was for dedicated software integrated and/or automation into clinical workflow was mentioned specifically in 7 (20.5%) responses.

## Service users’ perspective

### Q: When do you usually receive the ‘RECIST’ assessment? (tick all that apply)

A: As multiple responses to the question were allowed, a total of 45 responses were received from 35 respondents. 18 (40%) received measurements outcomes alongside the clinical report, 19 (42%) received them in addition to and after release of the clinical report although 6 (13%) respondents receive them in batches. Two responders chose to select the ‘other’ option, enabling a free text response detailing the need to *‘email/remind the named radiologist’*.

### Q: Is there a clear and defined process/workflow for the provision of ‘RECIST’ assessments in your institution? (tick all that apply)

A: Thirty-seven responses were provided from 35 responders. Clear and defined workflow documentation of the tumour measurement service for clinical trials was reported by 6 (16%) respondents, 4 (11%) reported this being provided on a trial-by-trial basis, 15 (41%) reported a clear process that was not documented but understood by all and 9 respondents (24%) described unclear local processes for provision of this work, 3 responders (8%) did not know.

### Q: How easy is it to get this measurement task fulfilled? (tick all that apply)

A: Thirty-six responses were returned from 35 responders. 12 (33%) explained that there were always people available to help, 12 (33%) reported that it could sometimes be difficult, 5 (14%) reported it as often difficult, 3 (8%) reported that it had been difficult in the past, 4 (11%) responders selected ‘other’, enabling a free text response highlighting that the requirement for a clinical report can delay the RECIST assessment and *‘it can be difficult as no-one is willing to do the extra work of RECIST’*.

### Q: Rate your service (1 to 10 with 1 being poor and 10 being excellent)

A: Two respondents did not answer. For timeliness the mean average score was 6.76 (median 7), for reliability the mean average score was 6.06 (median 6), 'Confidence in measurements and outcomes provided' scored better, with a mean average score of 7.7 out of 10 (median 9).

Thirteen of 34 (38.2%) respondents reported difficulties getting queries addressed. When asked to rate the service provided to them overall, the mean average score was 6.45 (median 7). A free text box was provided to allow respondents to briefly explain the reason for allotting the chosen score. The responses were varied but included *‘lack of resource, particularly radiologist time’*, issues with workflow causing delays including struggles associated with inconsistency and timeliness, especially *‘during holidays, strikes and winter pressures’*. There were, conversely, some positive comments including *‘a great team of engaged radiologists’, ‘excellent communication’* from *‘good, cancer focussed’* services.

## Discussion

Almost 9,000 oncology trials were registered in the UK between 1999 and 2022 [9], and the UK was the 6th highest recruiter into oncology trials worldwide during the same period [9]. The government response to Lord O’Shaughnessy’s report into commercial clinical trials [10] makes it clear that growth is a priority. It is remarkable therefore that this national survey indicates a lack of training, education, manpower and infrastructure to adequately support sustainable, good quality tumour response evaluation metrics which are critical oncology trial endpoints. The Department of Health and Social care policy paper ‘The future of clinical research delivery: 2022 to 2025’ [11] sets five themes for its 10-year vision – and relevant to this issue are specifically theme 1 focussing on a sustainable and supported research workforce, and theme 3 focussing on ease of access for patients across the UK to research. The findings of this survey demonstrate that there remains extensive ground to cover to address these and other challenges in response assessment in imaging.

A consistent feature of providers who were satisfied with the service they offer was the existence of local pathways for re-imbursement, with possible utilisation of the established tariff as detailed in the NIHR Schedule of Events Cost Attribution Template (SoECAT) [12]. This is likely to reflect the financial sustainability and effects on staff retention and recruitment that this enables. Only 29% of respondents indicated that they had robust financial reimbursement models in place. Ensuring transparency around costings and flow of funding for the work of NHS support services, such as Radiology, is essential to encourage investment in the appropriate infrastructure, staffing and training. This is being addressed by the NIHR Imaging National Speciality Group and guidance is being developed.

Many participants highlighted the need for affordable training, both to increase the capability of those currently providing the service but also to increase capacity. The lack of dedicated training for all disciplines (only 20% reported dedicated training provided) is of concern as is the low rates of regular audit (12%). The availability of standardised training and even accreditation for RECIST assessment may make sites more attractive to commercial sponsors, offering an assured high-quality service.

The survey indicated that despite workforce pressures, radiologists remain the primary providers of tumour response measurements (81%), with radiographers used much less commonly (10%). To meet local demands for reliable and effective imaging-based tumour response assessment, one model for service delivery is via developing a radiographer-led service [13]. Radiographer role extension for other applications is well established in the UK with 8 out of every 10 trusts in the UK using radiographers to report imaging [8] and demonstrating comparable results to radiologists in specific domains [14–16]. The use of a dedicated radiographer team to provide RECIST assessment can improve workflow and efficiency and ensure robust adherence to data governance and standard operating procedures [17]. In addition, role extension increases radiographer job satisfaction and aids recruitment and retention [18]. Our questionnaire highlighted a strong link between the sites using an accredited training course and an on-going audit and those that employed diagnostic radiographers in the service. This is almost certainly driven by the 2013 radiographer scope of practice guidance [19] which requires written confirmation in job description, education and training supplemented with regular audit and evidence of CPD.

The use of dedicated software to generate the results of the RECIST assessment showed some interest from those surveyed, with 9% of respondents already using a PACs-based or locally developed solution and a further 17% of respondents suggesting the use of such an approach as a development or improvement they would hope to see in the future. Such systems could offer efficiency savings and potential capacity increase but require validation as well as integration into existing clinical workflows.

The authors acknowledge the limitations of 69 responses, as well as a possible recruitment bias towards those already being supported to provide the service and/or those already engaged with the topic. However, responses have been received from a total of 35 institutions across the UK and, whilst, as expected, many major specialist oncology centres are represented, there were responses from some non-specialist centres. Some large, specialist centres reported ‘room for improvement’ in their services so it can only be assumed that smaller centres where funding and expertise is likely to be further stretched would be struggling to an even greater extent. There was no discernible link between the size of the institution and the service quality – the overriding factor affecting quality of service in this study pointed towards a local pathway of financial re-imbursement.

We suggest that there is a pressing need for UK training opportunities in response evaluation for clinical trials in addition to exemplars and templates for different models of delivery. Critical to success is establishment of local reimbursement direct to imaging departments to sustain and grow services. Together with the NIHR, the authors are assembling a ‘RECIST’ working group to collate and share best practice and training resources to aid sites build and develop a robust and sustainable service. There is an opportunity to develop this vital research service to support future growth of oncology trials in the UK. Future wider engagement with pharmaceutical partners will facilitate the development of a potential cost-effective and reliable model to service future clinical trials.

## Supplementary information

Supplementary information

## Data Availability

Anonymised survey responses have been supplied as supplementary material.
